# Post-Traumatic Headache is Associated with Worse Anxiety and Mood Symptoms in Adolescent Mild Traumatic Brain Injury

**DOI:** 10.31487/j.nnb.2022.02.04

**Published:** 2022-08-08

**Authors:** Michael W. Williams, Justin E. Karr, Alyssa M. Day, Amanda C. Glueck, Siddharth Kapoor, Dong Y. Han

**Affiliations:** 1 Department of Psychology, University of Houston, Houston, Texas, USA; 2 Department of Psychology, University of Kentucky, Lexington, Kentucky, USA; 3 Department of Neurology, University of Kentucky College of Medicine, Lexington, Kentucky, USA; 4 Department of Neurosurgery, University of Kentucky College of Medicine, Lexington, Kentucky, USA; 5 Department of Physical Medicine & Rehabilitation, University of Kentucky College of Medicine, Lexington, Kentucky, USA

**Keywords:** Anxiety, concussion, post-traumatic headache, adolescent, mental health, depression

## Abstract

This study examined the relationships between post-traumatic headache (PTH) and mental health symptoms after concussions to inform adolescent concussion management. Headache is the most common complaint following adolescent concussion. In this sample of 123 adolescents with concussion, there was a 5-fold increase in odds of clinically elevated anxiety, as well as increased mental health symptoms (anxiety, depression, anger, and disruptive behaviours), among adolescents with PTH relative to those without PTH. Adolescents with headache following concussions are vulnerable to worse mental health outcomes, particularly anxiety, and may benefit from routine monitoring of mental health symptoms for early detection and intervention.

## Introduction

Concussion, also referred to as mild traumatic brain injury (mTBI), is a common occurrence among adolescents in the United States, with nearly one in five adolescents between grades 8 and 12 reporting a previous diagnosis of concussion [1]. Although post-concussion symptoms can vary significantly, headache is often the most frequently endorsed symptom, followed by dizziness, distractibility, and confusion [2, 3]. Approximately 71–85% of adolescent patients report headache following a concussion or mTBI, making it the most common physical complaint from children and adolescents following injury [4–6]. In many instances, headache is associated with prolonged concussion symptoms [7].

Unrelated to concussion, psychological and mood disturbances are commonly reported among adolescents experiencing headaches [8]. Headaches are more common and frequent in those with anxiety and mood disorders, and adolescents with recurrent headaches are significantly more likely to report psychiatric comorbidity, such as anxiety or depression [9, 10]. Children and adolescents are significantly more likely to experience anxiety symptoms after a concussion compared to peers who sustained orthopaedic injuries [11]. Adolescents with a history of concussion are more likely to experience depressive symptoms compared to their peers, even after controlling for age, sex, socioeconomic status, and parental mental health [12, 13]. Further, adolescent girls and those with pre-injury psychiatric disorders are at greater risk for psychological distress following concussion [14, 15]. Thus, directionality between psychological problems and headache remains unclear at this point although studying PTH potentially can help elucidate this matter.

Due to the gender differences seen in the frequency of headaches and psychiatric diagnoses during adolescence, girls may be at particular risk for worse mental health symptoms following concussion. Girls are significantly more likely than boys to report headaches with and without a TBI [16–18]. Additionally, girls are at greater risk for depression and anxiety during adolescence compared to boys [19, 20]. However, findings on gender differences in mental health outcomes following concussion are scarce in the adolescent population [21]. While some evidence suggests girls are more likely to experience mental health symptoms following concussion; more research is needed to understand how mental health symptoms differ in adolescent boys and girls [14].

The prevalence of concussion in adolescent populations is high and subsequent headaches are common. Mental health status after concussion is a valuable outcome to examine, and symptoms may be related to the experience of PTH. With headache as the most common post-concussion symptom and frequent comorbidity with mental health problems, it is important to develop a better understanding of how headache following concussion may relate to mental health symptoms in adolescents [1, 5]. This study examined the following mental health domains: anxiety, depression, anger, disruptive behaviours, and self-concept. Further elucidation of relationships with PTH and specific mental health domains can enhance clinical decision-making in regard to symptom management and patient education. Given prior literature, it is expected that adolescents with PTH will demonstrate greater mental health symptoms following a concussion that are not attributable to gender differences. This study examined the differences in mental health symptoms after concussion between adolescents with and without PTH.

## Methods

### I Participants

There were 123 adolescents, ages 10 to 17, in this clinical dataset of patients evaluated at an academic medical center-based ambulatory concussion clinic between 2010 and 2012 eligible for this study. Out of the 174 adolescents in this dataset, 156 adolescents had valid data on headache status and all mental health outcomes. There were 33 patients excluded for missing time since injury data or for being outside the window of 14–365 days following concussion incident, leaving a final sample of 123.

### II Measures

#### i Demographics

Data regarding age, gender, race/ethnicity, years of education, and premorbid psychiatric disorder (clinical interview, self-report, and medical chart review for confirmation) were provided via patient self-report and medical chart review.

#### ii Premorbid Intelligence

The Wide Range Achievement Test, fourth edition (WRAT-4) Reading subtest was used to estimate premorbid intellectual functioning [22–24].

#### iii Injury Characteristics

Loss of consciousness was self-reported as no, yes, or probable; however, probable responses were treated as missing. Duration of loss of consciousness (self-report) was dichotomized to 0–30 minutes for mild severity and unknown [25]. No patients reported a duration greater than 30 minutes. Injury cause, based upon self-report and medical chart review, was categorized as a sport-related, motor vehicle collision, and other for this cohort.

#### iv Headache Status

Headache (no or yes) was assessed via clinical interview by the treating clinical neuropsychologist when reviewing presenting symptoms.

#### v Mental Health Symptoms

The Beck Youth Inventories for Children and Adolescents, second edition (BYI-II) consists of five self-report scales to measure anxiety, depression, anger, disruptive behaviour, and self-concept [26]. The anxiety scale reflects psychological worry and physiological symptoms associated with anxiety. The depression scale represents feelings of sadness, negative thoughts of self, life, or future, and physiological symptoms of depression. The anger scale captures feelings of anger, perceptions of mistreatment, and physiological arousal. The disruptive behaviour scale covers conduct problems. The self-concept scale depicts positive self-worth and perceptions of personal strengths. The BYI-II items ask about experiencing specific thoughts or feelings within the past two weeks. Response options are never, sometimes, often, or always. The BYI-II yields T-scores. Per the test manual, T-scores can be interpreted such that scores below 55 are average and scores of 55 and above are clinically elevated for anxiety, depression, anger, and disruptive behaviour scales. The self-concept scale interpretation indicates the T-scores below 45 are lower than average [26]. Adolescents completed the BYI-II independently in an ambulatory clinic setting.

### III Procedures

This study was conducted in accordance with the institutional review board guidelines (protocol # 46116). Data were collected as part of clinical care under the guidance of a clinical neuropsychologist. This study is a secondary analysis of a de-identified archival dataset.

### IV Data Analysis

All analyses were conducted using IBM SPSS v28 with an alpha level of 0.05. Data were screened for outliers and missing data. Differences in demographic characteristics by headache status were assessed using t-tests, χ^2^, and Fisher’s exact test. Significant differences in sociodemographic characteristics were considered for covariates in analyses. T-tests were used to examine mean differences in mental health symptoms (anxiety, depression, anger, disruptive behaviours, and self-concept) by headache status. The magnitude of effect size was evaluated with Cohen’s *d*. Chi-square and Fisher’s exact tests were used to examine differences in proportions of elevated BYI-II scores by headache status. Odds Ratios were calculated for effect size.

## Results

Participant descriptives are presented in (Table 1). The average age of the study sample was 14.7 years (*SD* = 1.7). The majority of participants were boys (65.9%). The mean years of education were 8.9 years. The most frequent cause of concussion was a sport-related incident (80.5%). Injury severity was in the mild range (loss of consciousness <30 minutes) for all known cases; however, there was a sizable number of cases (18.7%) with unknown duration of loss of consciousness. Of the sample (*n* = 123), the majority of adolescents endorsed PTH (*n* = 102; 82.9%), which is consistent with epidemiologic data [5].

A series of analyses were conducted to compare headache status groups on sociodemographic and injury-related characteristics. There were no differences between those with and without headaches with respect to age, gender, race, years of education, estimated premorbid intelligence, premorbid mental health disorder (no versus yes), mechanism of injury, presence or absence of loss of consciousness, duration of loss of consciousness (known mild range versus unknown), and days since injury (*p*s > .05). Interestingly, the proportion of girls with headache (90.5%) was greater than boys with headache (79.0%) although 82.9% of the overall sample endorsed headache, and boys represented majority of the total sample. However, this gender difference was not significant, *p* = .085. Also, the BYI-II T-scores are adjusted for gender and age.

Results of differences in mental health symptoms by headache status are presented in (Table 2). A Welch t-test is reported for the anxiety and anger scales due to homogeneity of variances being violated as assessed by Levene’s test for equality of variances (*p* < .05). However, all other mental health symptoms met the assumption of homogeneity of variances as determined by non-significant Levene’s test for equality of variances (*p* > .05). Participants who endorsed headache reported more anxiety symptoms than those who denied headache, *t*(50.20) = −4.89, *p* < .001, *d* = 0.95. The ratings of depression symptoms were higher for participants who endorsed headache compared to individuals without headache, *t*(121) = −2.48, *p* = .014, *d* = 0.63. Anger was higher among participants with headache compared to those who denied headache, *t*(47.41) = −4.53, *p* < .001, *d* = 0.90. Disruptive behaviours, similarly, were greater among participants with headache relative to participants without headache, *t*(121) = −3.71, *p* < .001, *d* = 0.97. Regarding self-concept, a positive resilience characteristic, individuals with headaches had slightly lower scores than individuals without headaches, but this difference was small and non-significant, *t*(121) = 1.24, *p* = .218, *d* = .29.

Evaluating differences in the proportion of clinically elevated scores (T ≥ 55, except for Self-Concept, T ≤ 44) revealed significant findings for the anxiety and anger scales. The headache status of adolescents was associated with clinically elevated anxiety scores (*X*^2^(1) = 5.09, *p* = .024). The Odds Ratio (OR) of clinically elevated anxiety was 4.97, indicating a higher probability among adolescents with headaches. The expected cell counts were lower than 5 for the remaining scales, precluding the use of chi-square test. As such, Fisher’s exact test was utilized. Clinical elevations on the anger scale were associated with positive headache status, *p* = .016. Among the adolescents with clinically elevated anger scores, 100% endorsed experiencing headaches (*n* = 20). However, headache was not associated with elevated depression scores (*p* = .194), elevated disruptive behaviour scores (*p* = .589), or clinically low self-concept scores (*p* = .124).

## Discussion

Among a sample of adolescent patients who presented to an outpatient concussion clinic, there were medium-to-large differences in the mental health symptoms between patients who did and did not report post-traumatic headache (PTH). Specifically, there were greater symptoms of anxiety, depression, anger, and disruptive behaviour among adolescent patients with PTH. In addition, when examining clinically elevated scores (T ≥ 55), PTH was associated with elevated anxiety and anger. However, there was no difference in self-concept, a potential measure of psychological resilience [27]. This study adds to the limited research knowledge on headaches after the pediatric concussion [28]. The differences in gender representation approached significance, with girls reporting slightly higher rates of headache than boys, although boys represented the majority of the sample. While this study did not find a meaningful level of gender difference, the direction of our findings is consistent with prior research. Among children and adolescents without a recent history of concussion, prior research has identified gender differences in headaches and non-localized pain [29, 30]. Within adolescents that have experienced a sport-related concussion, girls have reported greater headache severity than boys at their initial visit to an outpatient concussion clinic [31]. Adolescent girls tend to report greater post-concussion symptoms, including somatic and emotional [32]. However, although high school and college-aged girls tend to report greater severity of symptoms both before and after concussion, these differences, in aggregate, may not be clinically significant [33]. Importantly, this study utilized the BYI-II normative scores, which accounts for gender and age, as the measure for mental health symptoms.

The relationship between headache and specific mental health symptoms was clinically meaningful, with large group effects on symptoms of anxiety, anger, and disruptive behaviour and a medium group effect on symptoms of depression. These group differences could have functional significance, in that adolescents reporting headaches may be at greater risk for mental health problems and disruptive behaviours that may, but not always, interfere with academic achievement and lead to disciplinary issues at school [34, 35].

Interestingly, we found adolescents with PTH to have odds nearly five times that of adolescents without PTH for clinically elevated anxiety. The relationship between physical symptoms, such as headaches, and affective symptoms, such as depression and anxiety, are consistent with previous findings on post-concussion symptoms among adolescents. High correlations between physical and affective symptoms have been identified in prior factor analytic studies both prior to a concussion and following a concussion [36, 37].

Post-concussion symptoms are highly interrelated and interactive at baseline among student-athletes with mental health problems or attention-deficit/hyperactivity disorder [38, 39]. The potential unidirectional (e.g., frequent headaches → helplessness → depression and anger) or bidirectional (e.g. anxiety-related stress ↔ headaches) relationship between headaches and mental health problems can be easily conceptualized as an interactive symptom network [40]. If these concussion-related symptoms are interactive and mutually amplifying, then addressing each may ameliorate the other, which demonstrates the value of inter-professional concussion management by addressing headaches and mental health simultaneously. This dual management of TBI symptoms and pain using multiple approaches has been suggested by Mehalick and Glueck [41]. Dual management also acknowledges the complexities of concomitant challenges experienced by adolescents with concussions [42].

Of note, there were no differences in self-concept between participants with and without PTH, meaning the presence of headache does not equate to reduced psychological resilience. Positive perceptions of self could be valuable characteristics when addressing mental and behavioural health among adolescents recovering from concussion, including those with PTH. Although there is evidence to suggest psychotherapy is beneficial for addressing PTH among individuals with TBI, more studies with rigorous methodological standards are needed to make any definitive recommendations [43, 44]. This study indicates nuance with respect to emotional distress and psychological resilience among adolescents with concussion and PTH.

This study has several limitations, however. This clinical sample consists of adolescent patients who presented to an academic medical center ambulatory concussion clinic, which limits the generalizability of these findings to all adolescents with concussion. Many adolescents who experience a concussion may never present to a hospital-based clinic, receive treatment in another setting (e.g., through a school-based athletic trainer), or do not present for follow-up treatment. Our study is also limited by the absence of pre-injury symptom data as a baseline comparison, which is unfortunately common in such studies. The relationship between headaches and mental health symptoms may precede the injury for some patients in our sample and their headaches and their recent concussion may, or may not, amplify their mental health symptoms. Research has shown that pre-injury mental health disturbances are linked to worse concussion outcomes [45, 46]. As such, self-reported pre-injury psychiatric history information is included in (Table 1), and there was not a difference in rates of premorbid mental health conditions between headache groups. This lack of difference in premorbid psychiatric conditions across study groups strengthens the importance of these studies. The definition of headache was dichotomous and did not discriminate between different clinical headache subtypes, such as cervicogenic headaches, tension headaches, or migraines. Future research examining headaches and mental health following a concussion could specifically investigate whether different types of headaches, present both before and after injury, have differential relationships with mental health symptom ratings. Some prior investigations have found worse acute post-concussion outcomes associated with pre-injury migraines and worse subacute outcomes associated with PTH with migraine-like features [47–49].

Overall, the findings of this study indicated that PTH is a common and complicated problem for adolescent health. Adolescents experiencing PTH experienced greater mental health and behavioural problems, including greater anxiety, anger, depression, and disruptive behaviours, which were not attributable to gender differences in this study. A higher frequency of clinically elevated scores on anxiety and anger were associated with PTH. Although PTH is negatively related to mental health symptoms following concussion among adolescents, positive self-concept did not differ between individuals with and without headaches, which may represent psychological resilience in adolescents recovering from concussions. Considering these findings, it would be beneficial for medical providers engaged with adolescent health to routinely assess and monitor mental health symptoms among adolescents with PTH, as mental health symptoms and headache may have an interactive and bidirectional relationship. An assessment of mental health among adolescent patients with PTH can help to ensure that these patients recovering from concussions are connected with the appropriate interventions.

## Figures and Tables

**Table 1:** Sociodemographic characteristics of the study sample and subgroup comparisons.

Participant Characteristics	Total Sample *N* = 123	Headache		*p*
		No (*n* = 21)	Yes (*n* = 102)	

Age, *M*(*SD*)	14.7 (1.7)	14.6 (1.5)	14.7 (1.7)	.962
Gender	--			.085
Boys	81 (65.9%)	17 (81.0%)	64 (62.7%)	--
Girls	42 (34.1%)	4 (19.0%))	38 (37.3%)	--
Race	--			.433
White	120 (97.6%)	20 (95.2%)	100 (98.0%)	--
Black	3 (2.4%)	1 (4.8%)	2 (2.0%)	--
Years of education, *M*(*SD*)	8.9 (1.8)	8.9 (1.4)	8.9 (1.8)	.958
WRAT-4 Reading, *M*(*SD*)	103.3 (13.3)	103.0 (14.7)	103.4 (13.1)	.890
Premorbid mental health conditions	--			.079
No	104 (84.6%)	21 (100%)	83 (81.4%)	--
Yes	12 (9.8%)	0 (0.0%)	12 (11.8%)	--
Missing	7 (5.7%)	0 (0.0%)	7 (6.9%)	
Mechanism of Injury	--			.387
Sport-related	99 (80.5%)	16 (76.2%)	83 (81.4%)	--
Motor vehicle collision	8 (6.5%)	3 (14.3%)	5 (4.9%)	--
Fall	6 (4.9%)	1 (4.8%)	5 (4.9%)	--
Assault	2 (1.6%)	0 (0.0%)	2 (2.0%)	--
Other	6 (4.9%)	0 (0.0%)	6 (5.9%)	--
Missing	2 (1.6%)	1 (4.8%)	1 (1.0%)	
Duration of LOC	--			.542
30 minutes or less	100 (81.3%)	16 (76.2%)	84 (82.4%)	--
Unknown/missing	23 (18.7%)	5 (23.8%)	18 (17.6%)	--
Days since injury, *Mdn*(*IQR*)	38.0 (26.0–63.0)	44.0 (27.0–75.5)	35.0 (25.8–58.0)	.433

A t-test was used to examine group differences on age, education, and premorbid IQ (WRAT-4) variables. Fisher’s exact test was used to examine group differences on gender, race, premorbid mental health, and duration of loss of consciousness variables. Chi-square test was used to examine group differences on mechanism of injury. Mann-Whitney U test was used to examine group differences on days since injury variable.

*M*: Mean; *SD*: Standard Deviation; WRAT-4: Wide Range Achievement Test, Fourth edition; LOC: Loss Of Consciousness; *Mdn*: Median; *IQR*: Interquartile Range.

**Table 2:** Mental health symptoms by headache status. Psychiatric

Psychiatric Outcomes	Headache				*p*	*Cohen’s d*
	No		Yes			

	Mean	*SD*	Mean	*SD*		

Anxiety	44.1	5.5	51.8	9.8	<.001	0.95
Depression	43.2	7.0	48.1	8.4	.014	0.63
Anger	41.2	5.2	47.7	8.8	<.001	0.90
Disruptive Behavior	40.4	4.3	45.3	5.8	<.001	0.97
Self-Concept	53.9	9.2	51.3	8.3	.218	0.29

These outcomes are based upon scales from the Beck Youth Inventory, second edition. Anxiety reflects psychological worry and physiological symptoms associated with anxiety. Depression represents feelings of sadness, negative thoughts of self, life, or future, and physiological symptoms of depression. Anger captures feelings of anger, perceptions of mistreatment, and physiological arousal. Disruptive behaviour covers conduct problems. Self-Concept depicts positive self-worth and perceptions of personal strengths.
